# Correlation of disease activity in vitiligo patients and its relation with HSP70 expression

**DOI:** 10.6026/973206300221285

**Published:** 2026-02-28

**Authors:** Anup Kumar Mishra, Devesh Saraswat, Suneel Singh Sengar, Ayushi Jain, Sanjay Kumar Pandey

**Affiliations:** 1 Department of Dermatology, Shyam Shah Medical College and associated Hospitals, Rewa, Madhya Pradesh, India; 2 Multi-Disciplinary Research Unit, Shyam Shah Medical College Rewa, Madhya Pradesh, India

**Keywords:** Heat shock protein-70 (HSP-70), messenger RNA (mRNA), real-time PCR, vitiligo

## Abstract

An acquired pigmenting disorder, vitiligo, occurs worldwide with a prevalence of 0.5-2% and in India with 0.25%-4%. Stress-related pathways are involved may contribute to autoimmune melanocyte loss and HSP 70 (a stress-response molecular chaperone with immune- modulatory effects) may be a key link between stress and disease activity. In this case-control study, 40 vitiligo patients and 40 age and sex-matched controls underwent skin biopsies (lesional and non-lesional skin in patients; normal skin in controls) and HSP 70 mRNA expression was quantified using real-time PCR; patients were also grouped as highly active, moderately active or inactive based on clinical activity. Lesional vitiligo skin showed significantly higher HSP 70 expression than non-lesional skin and control skin (both P < 0.001), with higher mean levels in active compared with inactive vitiligo, while no association was found with age, sex or disease duration-supporting a role of HSP 70-related stress mechanisms in vitiligo pathogenesis and activity.

## Background:

Vitiligo is a chronic, progressive skin disorder characterized by melanocytes loss, leading to pigmented patches on the skin [1]. It occurs worldwide with a prevalence of 0.5-2% [2]. And in India it has a prevalence of 0.25%-4% [3]. Vitiligo in India is referred to as "shwetha- kustha" meaning white colored leprosy [4]. The disease has equal distribution among both genders and can appear at any age, although usually manifesting during childhood or early adulthood [5]. The origin of vitiligo is considered to be interaction of multiple factors, which includes genetic, autoimmune and environmental factors [6]. The pathogenesis of vitiligo remains poorly understood, with current research suggesting the involvement of immune-mediated destruction of melanocytes, oxidative stress and the role of various inflammatory mediators [7]. Disease activity, as defined by the rate of progression or stability of the depigmented lesions, is a significant factor in determining the course and prognosis of vitiligo [8]. Heat Shock Proteins (HSPs) are the proteins that are produced in response to stress and play a crucial role in cellular protection, repair and survival [9]. Among these, HSP-70 is one of the most widely studied, known for its cyto-protective functions against various stressors, including thermal, oxidative and inflammatory stress. It has been shown to participate in protein folding, degradation of damaged proteins and the regulation of apoptosis [10]. Therefore, it is of interest to investigate the correlation between HSP-70 expression and vitiligo disease activity to evaluate its potential as a biomarker for monitoring disease progression and stability.

## Materials and Methods:

## Ethics statement:

The study was conducted in accordance with the Declaration of Helsinki and was approved by the institute's ethics committee with approval number: 05/SS/MC/18 ON 26/02/2018. Informed written consent was obtained from all patients.

[1] Study setting: Study was conducted in institute's OPD.

[2] Study duration: 2 years.

[3] Study Population: Vitiligo patients between ages 12 to 60 years visiting institute's OPD who consented to participate in the study.

[4] Study design: case control study.

[5] Outcome variable: Level of HSP 70 mRNA expression in skin biopsies (lesional versus non lesional in vitiligo patients and normal skin in controls), quantitatively assessed via real time PCR and analysed in relation to disease activity.

## Consent:

All participants were informed about the purpose and the method of the research and the voluntary nature of participation in the study verbally as well as in written form and thereafter written consent were taken from the participants.

## Sample size:

This study included 40 vitiligo patients and 40 age and sex matched healthy controls presenting with the skin diseases in the Dermatology OPD.

## Participant selection:

## Inclusion criteria:

All clinically diagnosed vitiligo patients aged between 12 to 60 years and age & gender matched healthy controls that consented to participate in the study were included.

## Exclusion criteria:

Subjects (patients and controls) with malignancies, autoimmune diseases other than vitiligo and those receiving phototherapy, oral steroids or topical treatments in the past 6 months were also excluded from the study.

## Method:

Written informed consent was taken from all the participants coming to the OPD of dermatology. The complete history, physical and mucocutaneous examination was done for the exclusion of any associated diseases. Patient's details were taken including age, sex, type of vitiligo (generalized, localized or universal) and affected body surface area according to the rule of nines. Patients were classified into three groups in vitiligo disease: highly active vitiligo group (appearance of new lesions or progression of older lesion within the past 3 months), moderately active group (appearance of new lesions or progression of older lesion within the past >3 but <6 months) and inactive group (i.e., spontaneous improvement of existing lesions and or no appearance of new lesions or progression of older lesion within the past 6 months).

## Collection of samples:

Punch biopsies (with 3.5 mm punch) were taken from lesional and non-lesional skin of patients and from normal skin of controls. Detection of HSP70 messenger RNA was done using RT-PCR.

## Molecular investigation:

Every patient had 3.5 mm punch biopsies taken from their lesional and non-lesional skin, in addition to the controls' normal skin samples. Prior to the use of real-time polymerase chain reaction (RT-PCR) to identify the expression of HSP-70 messenger RNA (mRNA), all skin biopsies were kept at - 80°C.

## Detection of HSP-70 by RT-PCR:

Total RNA was isolated using the Resay Purification Reagent kit (Queen, Valencia, CA, USA) following the protocol recommended by the manufacturer. Using nanodrop, the purity (A260/A280 ratio) and concentration of RNA were determined (Nanodrop, iGene). Gel electrophoresis was used to verify the purity of the RNA.

## Cf DNA synthesis:

Using an Oligo (dT) 12-18 primer and Superscript II RNase Reverse Transcriptase, 4 µg of total RNA was reverse-transcribed into complementary DNA (ctDNA). This combination was then incubated at 42°C for one hour. The kit was provided by Superscript Choice System (Life Technologies, Breda and The Netherlands).

## RT-PCR:

The following primer sequence for the HSP-70 was found in 10 µL amplification mixtures employing SYBR Green PCR Master Mix (Hi-media, Mumbai, India), which was equivalent to 8 ng of reverse-transcribed RNA. Forward: 5'-AGCGT AACAC CACCA TTCC-3'; reverse: 5'-TGGCT CCCAC CCTAT CTC-3'. Reactions were run using a RT-PCR (Roche CobasZ 480) system. Data from the PCR reaction was analysed using sequence detection system software, which were conducted at 95°C for 10 min (one cycle), 94°C for 15 s and 60°C for 1 min (forty cycles). The comparative threshold cycle approach was used to calculate the relative expression of the genes under study with excel. The GAPDH genes served as the standard for all values. The forward primer 5'-AGC CAC ATC GCT GAG ACA C-3' and the reverse primer 5'-GCC CAA TAC GACCAA ATCC-3' comprised the GAPDH sequence.

## Statistical analysis:

Comparison of quantitative variables between the study groups was carried out using the Mann-Whitney U-test for independent samples. Data was entered in Microsoft excel 2007. P value <0.05 was considered significant.

## Results and Discussion:

Forty vitiligo patients aged 14-56 years (mean 33.12±12.67 years; 34 females, 6 males) and 40 age- and gender-matched healthy controls (mean 32.67±8.13 years; 32 females, 8 males) were included in the study. Among the patients, 22 (55%) had generalized vitiligo and 18 (45%) had localized vitiligo, with disease duration ranging from 1-80 months (Table 1). HSP-70 mRNA was expressed in skin biopsies of both patients and controls. However, the analysis revealed that the mean lesional HSP-70 mRNA level in vitiligo patients (0.62±0.18) was significantly higher than in controls (0.020±0.026; p < 0.001). Furthermore, non-lesional vitiligo skin also showed significantly higher levels (0.10±0.05) compared to controls (p < 0.001). Within the patient group, lesional levels were significantly higher than non-lesional levels (p < 0.001) (Table 2). These findings align with the autoimmune theory of vitiligo [11]. While the pathophysiology of vitiligo involves multiple hypotheses include genetic predisposition and impaired melanocyte adhesion [7]. Heat shock proteins (HSPs) have emerged as key contributors [8]. Normally, intracellular HSP-70 protects cells from apoptosis. However, melanocytes in vitiligo are prone to oxidative stress, which triggers the cellular stress response and the subsequent extracellular release of HSP-70 [12, 13]. Once released, extracellular HSP-70 can form complexes with melanocyte antigens. These complexes are recognized by dendritic cells, leading to T-cell activation, the release of pro-inflammatory cytokines (like IL-6, TNF-α) and subsequent melanocyte destruction [14, 15]. Our results are consistent with the study by Doss *et al.* which also found significantly increased HSP-70 expression in both lesional and non-lesional skin of vitiligo patients compared to healthy controls [16]. The elevation in non-lesional skin suggests that the entire skin surface in vitiligo patients may be under subclinical stress or immune dysregulation even before depigmentation becomes visible. When stratified by disease activity, a clear gradient in HSP-70 expression was observed. Lesional HSP-70 mRNA levels were highest in the highly active group (0.83±0.15; n = 12), followed by the moderately active group (0.74±0.06; n = 17) and lowest in the inactive group (0.46±0.20; n = 11). This association with activity was statistically significant (p < 0.01). A similar trend was observed in non-lesional skin (Table 3). The significantly elevated HSP-70 levels in active disease compared to inactive disease suggest that HSP-70 is actively involved in the progression of depigmentation. This supports findings by Denman *et al.* [17] and Moseson *et al.* [18], who demonstrated in mouse models that inducing HSP-70 expression (via vaccination) could drive depigmentation. The correlation suggests that higher oxidative stress and immune activation in active lesions result in greater HSP-70 release, perpetuating the autoimmune cycle. Similar associations of HSP-70 have been noted in other autoimmune conditions such as psoriasis (PS) and systemic lupus erythematosus (SLE) [19].

## Conclusion:

We show a significant correlation between vitiligo disease activity and the expression of Heat Shock Protein-70 (HSP-70). The findings support the potential use of HSP-70 as a biomarker for assessing disease activity and progression in vitiligo. Additionally, the results highlight HSP-70 as a potential therapeutic target, paving the way for further studies on developing the newer treatment modalities for vitiligo.

## Figures and Tables

**Table 1:** Baseline characteristics of study participants

Characteristic	Vitiligo patients	Healthy controls
Sample size, n	40	40
Age (years), mean±SD	33.12±12.67	32.67±8.13
Age range (years)	14-56	Not stated
Female sex, n (%)	34 (85.0)	32 (80.0)
Male sex, n (%)	6 (15.0)	8 (20.0)
Vitiligo type: generalized, n (%)	22 (55.0)	-
Vitiligo type: localized, n (%)	18 (45.0)	-
Disease duration (months)	1-80	-

**Table 2:** HSP-70 mRNA expression in skin biopsies

Outcome	Vitiligo patients (mean±SD)	Controls (mean±SD)	P value
HSP-70 mRNA level (lesional skin)	0.62±0.18	0.020±0.026	p < 0.001
HSP-70 mRNA level (non-lesional skin)	0.10±0.05	0.020±0.026	p < 0.001
Within-patient comparison (lesional versus non-lesional)	0.62±0.18 vs 0.10±0.05	-	p < 0.001

**Table 3:** Association of HSP-70 mRNA with disease activity

Disease activity group	n	Lesional HSP-70 mRNA (mean±SD)	Non-lesional HSP-70 mRNA (mean±SD)
Highly active	12	0.83±0.15	0.18±0.05
Moderately active	17	0.74±0.06	0.15±0.05
Inactive	11	0.46±0.20	0.12±0.05
